# Laboratory abnormalities and risk factors associated with in‐hospital death in patients with severe COVID‐19

**DOI:** 10.1002/jcla.23467

**Published:** 2020-07-12

**Authors:** Xing Chen, Li Yan, Yang Fei, Chi Zhang

**Affiliations:** ^1^ Department of Laboratory Medicine, Tongji Hospital, Tongji Medical College Huazhong University of Science and Technology Wuhan Hubei China; ^2^ School of Laboratory of Medicine Hubei University of Chinese Medicine Wuhan Hubei China

**Keywords:** cytokine storm, death, laboratory parameter, risk factors, the severe COVID‐19

## Abstract

**Background:**

In the context of the COVID‐19 outbreak of worldwide, we aim to analyze the laboratory risk factors of in‐hospital death in patients with severe COVID‐19.

**Methods:**

All ≥18‐year‐old patients with confirmed severe COVID‐19 admitted to Tongji Hospital (Wuhan, China) from February 3 to February 20, 2020, were retrospectively enrolled and followed up until March 20, 2020. Epidemiological, clinical, laboratory, and treatment data were collected and explored the risk factors associated with in‐hospital death.

**Results:**

A total of 73 severe patients were enrolled in the study, of whom 20 (27%) patients died in hospital during the average 28 days of follow‐up period. The median age of non‐survivors was significantly older than survivors (69 [64‐76.5] years vs 64 [56‐71.3] years, *P* = .033) and 15 (75%) patients were males. The laboratory abnormalities of non‐survivors mainly presented in serious inflammation response and multiple organ failure, with high levels of cytokines and deranged coagulation parameters. Multivariable regression showed that neutrophil count greater than 4.47 × 10^9^/L (OR, 58.35; 95%CI: 2.16‐1571.69; *P* = .016), hypersensitivity C‐reactive protein greater than 86.7 mg/L (OR, 14.90; 95%CI: 1.29‐171.10; *P* = .030), creatine kinase greater than 101 U/L (OR, 161.62; 95%CI: 6.45‐4045.20; *P* = .002), and blood urea nitrogen greater than 6.7 mmol/L (OR, 11.18; 95%CI: 1.36‐91.62; *P* = .024) were risk factors for in‐hospital death.

**Conclusion:**

The risk factors of neutrophil count, hypersensitivity C‐reactive protein, creatine kinase, and blood urea nitrogen could help clinicians to early identify COVID‐19 severe patients with poor outcomes on admission. Virus direct attack and cytokine storm play a major role in the death of COVID‐19.

## Graphical abstract

Neutrophil count, hypersensitivity C‐reactive protein, creatine kinase, and blood urea nitrogen were risk factors for in‐hospital death in patients with severe COVID‐19. Virus direct attack and cytokine storm play a major role in fatal outcomes.

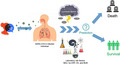

**Abbreviations**
ACE2: angiotensin I converting enzyme 2
ARDS: acute respiratory distress syndrome
BUN: blood urea nitrogen
CK: creatine kinase
COVID‐19: Coronavirus disease 2019
DIC: disseminated intravascular coagulation
ESR: erythrocyte sedimentation rate
FiO2: fraction of inspired oxygen
GFR: glomerular filtration rate
hs‐CRP: hypersensitivity C‐reactive protein
hs‐cTnI: hypersensitive cardiac troponin I
LDH: lactate dehydrogenase
LYM: lymphocyte count
NEU: neutrophil count
NT‐proBNP: N‐terminal pro‐brain natriuretic peptide
PaO2: arterial partial pressure of oxygen
PT: prothrombin time
ROC: the receiver operating characteristic
SARS‐COV‐2: severe acute respiratory syndrome coronavirus 2
WBC: white blood cell count

## 1 INTRODUCTION

The novel coronavirus disease 2019 (COVID‐19) is a systemic illness with the respiratory system as the main symptom caused by severe acute respiratory syndrome coronavirus 2(SARS‐COV‐2), which belongs to the Coronaviridae family.^1^, ^2^ Since the first case was reported in Wuhan, China, in December 2019, the virus had quickly swept the global, and as of May 14, 2020, about 4 248 389 confirmed cases, with as many as 292 046 related deaths have been officially reported worldwide.^3^ COVID‐19 can cause mild‐to‐severe illness, and serious illness occurs in 15.7% of the patients in hospitalization.^4^ Unfortunately, there is currently no specific antiviral drug against the virus. Therefore, many patients with severe type suffered a death attack in the hospital due to a relative lack of effective treatment.

Although previous studies had fully described the clinical characteristic of COVID‐19 in severe and death cases, research mainly aimed at laboratory abnormalities found in patients with COVID‐19 from severe cases to death cases is still lacking. There is no doubt that laboratory medicine plays an utmost part in the detection and identity the virus, in which the positive nucleic acid testing had been one of the golden standards in clinical diagnosis COVID‐19.^5^ Nevertheless, the laboratory parameter could also be used for predicting disease progression and assessing prognosis.

Here, we present the demographic, clinical, laboratory, and treatment data of hospitalized patients between survivors and non‐survivors, describe the main laboratory abnormalities, and analyze the risk factors of in‐hospital death with the hope of reducing mortality.

## 2 METHODS AND MATERIALS

### 2.1 Study design and participants

This retrospective study was done at three branches of Tongji hospital, which are located 14 km to 34 km from one another in central Wuhan. We enrolled consecutive hospitalized adult patients (≥18 years old) admitted from February 3 to February 20, 2020, who were laboratory‐confirmed COVID‐19 and categorized as severe type based on the clinical presentation at the time of admission. All patients were diagnosed with COVID‐19 according to WHO interim guidance. The severe type was defined if satisfying at least one of the following criteria: respiratory rate ≥30 times/min, resting oxygen saturation ≤93%, and arterial partial pressure of oxygen (PaO_2_)/fraction of inspired oxygen (FiO_2_) ≤300 mm Hg. Patients were classified into survivors and non‐survivors during the average 28 days of follow‐up period. For patients who were alive by February 20, 2020, their living status was confirmed on March 20, 2020. To minimize interference in analyzing the risk factors associated with in‐hospital death, non‐survivors who died within 3 days after admission were excluded. The study was approved by the Research Ethics Commission of Tongji Hospital (Wuhan, China). All data were analyzed anonymously.

### 2.2 Data collection

Epidemiological, clinical, laboratory, and treatment data were extracted from electronic medical records. The clinical data used in this study were collected from the initial evaluations of hospital admission. All data were checked by two physicians (Xing Chen and Li Yan), and a third researcher (Chi Zhang) adjudicated any difference in interpretation between the two primary reviewers.

### 2.3 Statistical analysis

Continuous variables were defined as the median (interquartile range) for non‐Gaussian distribution data and compared with the Mann‐Whitney U test; categorical variables were presented as numbers and percentages (%) and compared by Fisher's exact test between survivors and non‐survivors. Spearman correlation coefficient was calculated between different parameters for non‐parametric correlations. The cutoff value was selected when the Youden index of the receiver operating characteristic (ROC) curve by a logistic regression model was the largest. Univariable and multivariable logistic regression analyses were performed to explore the risk factors associated with in‐hospital death. Statistical analyses were performed by SPSS 22.0 (IBM Corporation), and a two‐sided *P* < .05 was statistically significant.

## 3 RESULTS

From February 3, 2020, to February 20, 2020, a total of 105 adult COVID‐19 patients were recorded in Tongji Hospital and categorized into severe type when admitted. After excluded 8 cases that died within 3 days and 24 cases due to incomplete medical records, we finally enrolled 73 severe patients. At the end of the study, 20 (27.4%) patients had died in the hospital, and the median time from admission to death was 12.0 days (IQR, 6.25‐17.5). Of the 53 patients who survived, 17 (32.1%) patients were discharged and the median time from admission to discharge was 21.9 days (IQR, 17‐27).

The median age of all patients was 66 years (IQR: 59‐72.3), and 42 (57.5%) patients were male. On admission, the most common symptoms were fever (94.5%), cough (57.5%), dyspnea (31.5%), and diarrhea (27.4%). Other less common symptoms were chest tightness (17.8%), tachypnea (13.7%), and fatigue (13.7%). A total of 57 patients had comorbidities, with hypertension (45.2%) is the most common ones, followed by diabetes (20.5%) and cardiovascular disease (12.3%). Neither common symptoms nor comorbidity showed significantly different between survivors and non‐survivors, except the age (*P* = .033). All patients received antivirals and 69 (94.5%) patients received antibiotics. Non‐invasive (*P* < .001) and invasive (*P* < .001) mechanical ventilation were significantly different between two groups, detailed data are showed in Table 1.

**Table 1:** Baseline information of COVID‐19 severe patients enrolled in this study

	Total	Survival	Non‐survival	*P* value
N	73	53	20	\
Age, y	66 (59.00‐72.30)	64 (56.00‐71.30)	69 (64.00‐76.50)^a^	.033
Males	42 (57.5%)	27 (50.9%)	15 (75.0%)	.109
Duration from onset to admission, d	10 (7.5‐12)	11.0 (8.3‐13.0)	12 (10.0‐13.0)	.442
Time from admission to death, d	\	\	12 (6.25‐17.5)	\
Initial symptoms				
Fever	69 (94.5%)	49 (92.5%)	20 (100.0%)	.569
Cough	42 (57.5%)	27 (50.9%)	15 (75.0%)	.109
Sputum production	3 (4.1%)	2 (3.8%)	1 (5.0%)	1.000
Dizziness	3 (4.1%)	2 (3.8%)	1 (5.0%)	1.000
Chest tightness	13 (17.8%)	9 (17.0%)	4 (20.0%)	.742
Myalgia	6 (8. 2%)	4 (7.5%)	2 (10.0%)	.663
Tachypnea	10 (13.7%)	8 (15.1%)	2 (10.0%)	.717
Diarrhea	20 (27.4%)	14(26.4%)	6 (30.0%)	.774
Fatigue	10(13.7%)	6 (11.3%)	4 (20.0%)	.446
Dyspnea	23 (31.5%)	20 (37.7%)	3 (15.0%)	.090
Comorbidities				
Hypertension	33 (45.2%)	23 (43.4%)	10 (50.0%)	.792
Diabetes	15 (20.5%)	13 (24.5%)	2 (10.0%)	.211
Cardiovascular disease	9 (12.3%)	6 (11.3%)	3 (15.0%)	.698
Lung disease	7 (9.6%)	6 (11.3%)	1 (5.0%)	.665
Cerebral infarction	3 (4.1%)	2 (3.8%)	1 (5.0%)	1.000
Chronic liver disease	2 (2.7%)	2 (3.8%)	0 (0.0%)	1.000
Malignant tumor	6 (8.2%)	4 (7.5%)	2 (10.0%)	.663
Treatments				
Antibiotics	69 (94.5%)	49 (92.5%)	20 (100%)	.570
Antiviral Chinese Herb	67 (91.8%)	48 (90.6%)	19 (95%)	1.000
Antiviral modern drug	73 (100%)	53 (100%)	20 (100%)	1.000
Corticosteroids	62 (84.9%)	43 (81.1%)	19 (95%)	.270
Immunoglobin	35 (47.9%)	22 (41.5%)	13 (65%)	.114
High flow nasal cannula	70 (95.9%)	51 (96.2%)	19 (95%)	1.000
Non‐invasive mechanical ventilation	33 (45.2%)	13 (24.5%)	20 (100%)^a^	<.001
Invasive mechanical ventilation	18 (24.7%)	7 (13.2%)	11 (55%)^a^	<.001

Abbreviations: COVID‐19, coronavirus disease 2019; IQR, interquartile range; N, number.

Continuous variables were presented as median and IQR; categorical variables were given as number and percentages; p values were calculated by Mann‐Whitney U test or Fisher's exact test.

^a^ Compared with COVID‐19 survival group, *P* < .05.

There was quite a lot of difference in laboratory parameters between the two groups. Compared to survivors, non‐survivors demonstrated significantly reduced lymphocyte count (LYM) (0.51 vs 0.73 × 10^9^/L) and increased neutrophil count (NEU) (6.89 vs 4.47 × 10^9^/L) on admission. Inflammation‐related marker include hypersensitivity C‐reactive protein (hs‐CRP) (168.9 vs 51.1 mg/L), procalcitonin (0.16 vs 0.07 ng/mL), and serum ferritin (1375 vs 1140 μg/L) were higher in non‐survivor patients, except for erythrocyte sedimentation rate (ESR) (42 vs 43 mm/60 min). For coagulation function, non‐survivors showed prolonged prothrombin time (PT) (15.6 vs 14.2 seconds) and elevated level of D‐dimer (9.68 vs 1.13 μg/mL). Besides, the level of cytokines (interleukin‐6, interleukin‐8, interleukin‐10, and interleukin‐2 receptor) in non‐survivor patients was increased except for interleukin‐1β (5 vs 5 pg/mL). The organ damage biomarkers hypersensitive cardiac troponin I (hs‐cTnI)(32.3 vs 7.2 pg/mL), N‐terminal pro‐brain natriuretic peptide (NT‐proBNP) (794 vs 301 pg/mL), creatine kinase (CK)(127 vs 52 U/L), and blood urea nitrogen (BUN)(9.0 vs 5.0 mmol/L) also increased compared with survivor patients. Laboratory examination result details are showed in Table 2.

**Table 2:** Laboratory examination of COVID‐19 severe patients enrolled in this study

	Total	Survival	Non‐survival	*P*
n	73	53	20	\
WBC, × 10⁹/L	6.78 (4.99‐9.15)	6.34 (4.63‐8.79)	7.79 (6.33‐9.93)^a^	.022
NEU, × 10⁹/L	5.51 (3.86‐7.64)	4.47 (3.22‐6.84)	6.89 (5.58‐9.09)^a^	.002
LYM, × 10⁹/L	0.69 (0.50‐0.96)	0.73 (0.56‐1.31)	0.51 (0.38‐0.73)^a^	.002
PT, s	14.5 (13.9‐15.6)	14.2 (13.7‐14.8)	15.6 (14.8‐17.3)^a^	.000
APTT, s	40.2 (34.6‐44.7)	42.0 (36.0‐45.4)	34.8 (33.7‐40.0)^a^	.006
Fibrinogen, g/L	5.47 (4.15‐6.31)	5.47 (4.24‐6.18)	5.51 (3.42‐6.79)	.711
D‐dimer, μg/mL	1.30 (0.88‐6.32)	1.13 (0.76‐2.25)	9.68 (1.82‐21.00)^a^	.000
hs‐CRP, mg/L	65.3 (28.7‐134.5)	51.1 (20.4‐86.3)	168.9 (90.2‐220.8)^a^	.000
Serum ferritin, μg/L	1326 (762‐1703)	1140 (630‐1523)	1375 (1000‐2135)	.056
ESR, mm/60min	43 (22.5‐63.5)	43 (22.5‐60)	42 (22‐68.7)	.833
Interleukin‐1β, pg/mL	5 (5‐5)	5 (5‐5)	5 (5‐5)	.739
Interleukin‐2 receptor, U/mL	941 (658‐1274)	800 (638‐1145)	1030 (942‐1496)^a^	.011
Interleukin‐6, pg/mL	35.5 (13.2‐81.9)	27.2 (12.6‐62.0)	84.3 (22.6‐137.8)^a^	.011
Interleukin‐8, pg/mL	24.3 (16.0‐52.0)	19.5 (13.1‐45.3)	47.5 (20.2‐101.0)^a^	.003
Interleukin‐10, pg/mL	8.3 (5.0‐11.9)	6.7 (5.0‐10.3)	11.1 (5.8‐16.6)^a^	.031
TNF‐α, pg/mL	9.1(7.6‐11.2)	9.1 (7.3‐11.1)	9.9 (7.9‐21.2)	.165
hs‐cTnI, pg/mL	10.2 (5.3‐22.5)	7.2 (5.0‐15.2)	32.3 (10.3‐176)^a^	.000
NT‐proBNP, pg/mL	359 (174‐1056)	301 (153‐555)	794 (256‐1467)^a^	.010
Procalcitonin, ng/Ml	0.08 (0.05‐0.15)	0.07 (0.05‐0.09)	0.16 (0.10‐0.96)^a^	.000
Glucose, mmol/L	6.92 (5.97‐9.24)	6.61 (5.75‐8.85)	7.16 (6.34‐13.50)	.168
ALT, U/L	28 (17‐41)	27 (16‐41)	30 (20.5‐56.5)	.325
AST, U/L	35 (25‐57.5)	33 (24.5‐56)	38 (30‐68.3)	.310
CK, U/L	65 (41.5‐109)	52 (38‐75)	127 (78.75‐323.8)^a^	.000
LDH, U/L	407 (265‐520)	347 (261‐482)	491 (378‐660)^a^	.031
Creatinine, μmol/L	72 (54‐89)	69 (52‐82)	85 (72‐114)^a^	.006
BUN, mmol/L	5.6 (3.9‐7.9)	5.0 (3.5‐6.5)	9.0 (5.82‐13.0)^a^	.000
GFR, mL/min/1.73 m^2^	90.7 (74.3‐98.9)	92.3 (80.3‐101.2)	83.8 (45.5‐97.3)^a^	.035

Abbreviations: ALT, alanine transaminase; APTT, activated partial thromboplastin time; AST, aspartate aminotransferase; BUN, blood urea nitrogen; CK, creatine kinase; COVID‐19, coronavirus disease 2019; ESR, erythrocyte sedimentation rate; GFR, glomerular filtration rate; hs‐CRP, hypersensitivity C‐reactive protein; hs‐cTnI, hypersensitive cardiac troponin I; IQR, interquartile range; LDH, lactate dehydrogenase; LYM, lymphocyte count; N, number; NEU, neutrophils count; NT‐proBNP, N‐terminal pro‐brain natriuretic peptide; PT, prothrombin time; TNF, tumor necrosis factor; WBC, white blood cell count.

Continuous variables were presented as median and IQR; categorical variables were given as number and percentages; *P* values were calculated by Mann‐Whitney U test.

^a^ Compared with COVID‐19 survival group, *P* < .05.

Next, Spearman correlation analysis showed that white blood cell count (WBC) was positively correlated with NEU (*r* = .958); creatinine was positively correlated with BUN, while negatively correlated with GFR (*r* = .705, *r* = −.759, respectively). Therefore, WBC and creatinine were excluded in logistic regression model due to its high correlation with other parameters.

In the final multivariable logistic regression model, we found that increased NEU (OR: 58.35; 95%CI: 2.16‐1571.69; *P* = .0155), hs‐CRP (OR: 14.90; 95%CI: 1.27‐171.10; *P* = .0300), CK (OR: 161.62; 95%CI: 6.45‐4045.20; *P* = .0020), and BUN (OR: 11.18; 95%CI: 1.36‐91.62; *P* = .0244) were independent risk factor of COVID‐19 in‐hospital death. The detailed results of univariable and multivariable regression are shown in Table 3.

**Table 3:** Risk factors associated with COVID‐19 in‐hospital death

	Univariable OR(95%CI)	*P* value	Multivariable OR(95%CI)	*P* value
Age, y (vs ≤ 61)	4.02 (1.04‐15.41)	.0423		
NEU,×10^9^/L (vs ≤ 4.47)	19.73 (2.46‐158.21)	.0050	58.35 (2.16‐1571.69)	.0155
LYM,×10^9^/L (vs > 0.55)	6.34 (2.06‐19.47)	.0012		
PT, s (vs ≤ 14.7)	15.78 (4.00‐62.17)	.0001		
APTT, s (vs >40.2)	6.09 (1.78‐20.77)	.0039		
D‐dimer, μg/mL (vs ≤ 2.35)	10.25 (3.08‐34.00)	.0001		
hs‐CRP, mg/L (vs ≤ 86.7)	13.66 (3.83‐48.69)	.0001	14.90 (1.29‐171.10)	.0300
IL‐2R,U/mL (vs ≤ 867)	7.98 (2.08‐30.60)	.0024		
Interleukin‐6, pg/mL (vs ≤ 81.98)	9.57 (2.81‐32.55)	.0003		
Interleukin‐8, pg/mL (vs ≤ 46.4)	5.25 (1.71‐16.07)	.0036		
Interleukin‐10, pg/mL (vs ≤ 8.6)	4.53 (1.49‐13.80)	.0077		
hs‐cTnI, pg/mL (vs ≤18.9)	8.43 (2.62‐27.15)	.0003		
NT‐proBNP, pg/mL (vs ≤ 570)	5.12 (1.70‐15.42)	.0037		
Procalcitonin, pg/mL (vs ≤ 0.09)	15.27 (4.24‐54.99)	.0001		
CK, U/L (vs ≤101)	18.27 (5.08‐65.69)	<.0001	161.62 (6.45‐4045.20)	.0020
LDH, U/L (vs ≤ 450)	5.39 (1.75‐16.56)	.0032		
BUN, mmol/L (vs ≤ 6.7)	10.03 (3.08‐32.59)	.0001	11.18 (1.36‐91.62)	.0244
GFR, mL/min/1.73 m^2^ (vs > 63.1)	6.40 (1.77‐23.11)	.0046		

Abbreviations: APTT, activated partial thromboplastin time; BUN, blood urea nitrogen; CK, creatine kinase; COVID‐19, coronavirus disease 2019; GFR, glomerular filtration rate; hs‐CRP, hypersensitivity C‐reactive protein; hs‐cTnI, hypersensitive cardiac troponin I; IL‐2R, interleukin‐2 receptor; LDH, lactate dehydrogenase; LYM, lymphocyte count; NEU, neutrophil count; NT‐proBNP, N‐terminal pro‐brain natriuretic peptide; OR (95%CI), odds ratio(95% confidence interval); PT, prothrombin time; WBC, white blood cell count.

OR and *P* values were calculated by logistic regression.

## 4 DISCUSSION

In this retrospective cohort study, we reported the risk factors associated with in‐hospital death and main laboratory abnormalities in COVID‐19 severe patients. We found that severe COVID‐19 patients having higher NEU (above than 4.47 × 10^9^/L), hs‐CRP (above than 86.7 mg/L), CK (above than 101 U/L), and BUN (above than 6.7 mmol/L) on admission faced greater risk in‐hospital death. Besides, the laboratory alternations in non‐survivors mainly focused on the prominent inflammation response and multiple organ damage, with the increased levels of cytokines and dysregulation of coagulation parameters.

SARS‐COV‐2 infection predominantly infects lower airways and causes severe pneumonia, which sometimes results in acute respiratory distress syndrome (ARDS) and progressive respiratory failure.^6^, ^7^ In our study, 70 (95.9%) patients required high flow nasal cannula and all non‐survivors received non‐invasive mechanical ventilation. Since no specific antiviral drug to treat the SARS‐COV‐2 at present, the mainstay of clinical treatment has been supportive care and the effect of clinical therapeutic is poor. Although a previous study has reported that the mortality rate of COVID‐19 patients is 1.4%,^4^ but the in‐hospital death of patients with severe type COVID‐19 was 27% in our study, which is similar to the reports of Zhou et al^8^ And another report showed the mortality was 61.5% among critically ill patients.^9^ This message might reflect the mortality is high among COVID‐19 severe patients in the stage of the outbreak, meaning that clear out the risk factors of death is crucial to control the rising number of deaths.

Our study first focused on the baseline information of COVID‐19 severe patients. The median age of non‐survivors was significantly older than survivors (69 [64‐76.5] years vs 64 [56‐71.3] years, *P* = .033) and most of them (75%) were male. As for common symptoms of illness onset, we discovered that excluded fever and cough, 30% of non‐survivor cases expressed diarrhea. Patients with digestive symptoms may be ignored if clinicians only or mainly focus on respiratory symptoms. The comorbidity is an essential component in determining the disease progress^10^; however, there is no difference in underlying diseases between two groups in our study even though most of them have one or more comorbidities. Probably because they affect the disease progression of patients with mild or moderate type.^4^ In general, as mentioned in previous studies,^4^, ^8^, ^9^ older adult males are more likely to exhibit worse outcomes due to weak immune functions in this population.

Previously, similar to SARS, lung damage has been reported not the only cause of death in COVID‐19, but also include heart, liver, kidney, and other organ dysfunction.^11^, ^12^ Our study also showed that BUN(9.0 vs 5.0 mmol/L, *P* < .01), glomerular filtration rate (GFR)(82.8 vs 92.3 mL/min/1.73 m^2^, *P* = .035), creatinine (85 vs 69 μmol/L, *P* = .06), NT‐proBNP (794 vs 301 pg/mL, *P* = .01), hs‐cTnI (32.3 vs 7.2 pg/mL, *P* < .01), and CK(127 vs 52 U/L, *P* < .01) have significance differences among two groups. Especially BUN and CK, which within the reference range in survivor patients and above the normal range in non‐survivor patients, providing superior clinical significance in predicting the odds of in‐hospital death on admission. Considering that COVID‐19 and SARS share the same receptor angiotensin I converting enzyme 2 (ACE2) to invade host cells, Zhou et al^13^ analyze the expression of ACE2 in human organs. The study showed that the kidney, heart, and liver have portion ACE2 positive cells, suggesting the virus travels follow the blood and directly attack the targeting organ, resulting in organ function damage and even multiple organ failure.

Other laboratory abnormalities expressed in the hyperinflammatory response include the elevated inflammatory markers hs‐CRP (168.9 vs 51.1 mg/L), serum ferritin (1375 vs 1140 μg/L), and procalcitonin (0.16 vs 0.07 ng/mL), except for ESR (42 vs 43 mm/60 min). The increased procalcitonin and hs‐CRP are strongly suggestive of secondary bacterial infection, as bacteria attack the already fragile immune system. In multivariable regression analysis, we showed that patients had poor prognosis when hs‐CRP greater than 86.7 mg/L.

The immunopathological mechanism of SARS‐COV and MERS‐COV remains unclear, but the correlative evidence suggested that the persistent production of enormous cytokines known as “cytokine storms” play a crucial role in immune hyperactivity.^14^ Compared to other reports, our study analyzed the differences of major cytokines between two groups. Significantly high serum levels of interleukin‐6, interleukin‐8, interleukin‐10, and interleukin‐2 receptor could be observed in non‐survivors, which implying the cytokine storms associated with the poor outcomes in COVID‐19. Interestingly, the concentration of interleukin‐1β (5 vs 5 pg/mL, *P* = .739) was no difference between the two groups. The reason may be that interleukin‐1β showed as early response cytokine to viral infection, whereas our cases were in the late inflammation.^15^ Cytokines and chemokines produced by virus‐infected cells not only resulting in the extensive infiltration of neutrophils and macrophages but also lead to tissue damage, both locally and systemically. Besides, the accumulating NEU secretes myeloperoxidase and elastase to directly destroy the lung structure.^14^, ^15^ These results, consistent with the previous findings, suggest that the death of COVID‐19 may be attributable to the “cytokine storms” and virus direct attack.^11^, ^14^, ^15^

Moreover, the accumulating neutrophils and macrophages themselves produce additional cytokines and chemokines, which in turn enhance disease severity.^14^ In this study, when the absolute number of circulating NEU is greater than 4.47 × 10^9^/L, severe COVID‐19 cases would face a greater risk of in‐hospital death. Perhaps, increased NEU also plays a crucial role in COVID‐19 immunopathology. Further research should be undertaken to investigate the role of NEU in cytokine storm when SARS‐COV‐2 infection.

Our study also reported the disturbed coagulation status of non‐survivor patients. Disseminated intravascular coagulation (DIC) is a persistent activation of the hemostatic system mainly caused by infection, inflammation, and surgery.^16^ A recent study showed that COVID‐19 severe patients have ischemic changes in the fingers and may develop DIC.^17^, ^18^ In our study, significantly prolonged PT (15.6 vs 14.2 s, *P* = .000) and increased D‐dimer (9.68 vs 1.63 μg/mL, *P* = .000) suggested an excessive coagulation activation in non‐survivor patients. Therefore, some researchers proposed for low‐molecular‐weight heparin anticoagulant therapy in patients with severe COVID‐19.^17^

There are several limitations in our study. First, not all laboratory parameters demonstrated here, but our results can still be considered useful for most hospital clinicians. Second, the lack of definite outcomes for some survivors may affect the observation of the disease. Last, as a relatively small, single‐center retrospective study, only 73 patients were included in this study, which may not be representative; further information from a larger cohort would help to better define the risk factors of in‐hospital death.

In conclusion, virus direct attack and cytokine storm are believed to play a powerful role in deleterious consequences. Our study reported that elevated levels of NEU, hs‐CRP, CK, and BUN were risk factors for in‐hospital death in COVID‐19 severe patients.

## AUTHOR CONTRIBUTIONS

Xing Chen, Li Yan, and Chi Zhang contributed to the intellectual content of this paper and drafted and revised the manuscript. Yang Fei contributed to collection and analysis of the experiment data. All the authors have read and accepted the final version of the manuscript and approved its submission.

## ETHICS APPROVAL

The study protocol was approved by the Tongji Hospital Ethics Committee for Research in Health.
